# Social and physical environment independently affect oviposition decisions in *Drosophila*

**DOI:** 10.1093/beheco/arab105

**Published:** 2021-09-22

**Authors:** Emily R Churchill, Calvin Dytham, Jon R Bridle, Michael D F Thom

**Affiliations:** 1 School of Biological and Marine Sciences, University of Plymouth, Plymouth, UK; 2 Department of Biology, University of York, York, UK; 3 Department for Genetics, Evolution and Environment, University College London, London, UK

**Keywords:** competition, copulation, density, Drosophila melanogaster, egg laying, mating duration, patchiness, resource distribution

## Abstract

In response to environmental stimuli, including variation in the presence of conspecifics, genotypes show highly plastic responses in behavioral and physiological traits influencing reproduction. Although extensively documented in males, such female responses are rather less studied. We expect females to be highly responsive to environmental variation and to differentially allocate resources to increase offspring fitness, given the major contribution of mothers to offspring number, size, and developmental conditions. Using *Drosophila melanogaster*, we (a) manipulate exposure to conspecific females, which mothers could use to anticipate the number of potential mates and larval density, and; (b) test how this interacts with the spatial distribution of potential oviposition sites, with females from higher densities expected to prefer clustered resources that can support a larger number of larvae. We found that high density females were slower to start copulating and reduced their copulation duration, the opposite effect to that observed in males. There was a parallel, perhaps related, effect on egg production: females previously housed in groups laid fewer eggs than those housed in solitude. Resource patchiness also influenced oviposition behavior: females preferred aggregated substrate, which attracted more females to lay eggs. However, we found no interaction between prior housing conditions and resource patchiness, indicating that females did not perceive the value of different resource distributions differently when exposed to environments that could signal expected levels of larval competition. We show that, although exposure to consexual competition changes copulatory behaviors of females, the distribution of oviposition resources has a greater effect on oviposition decisions.

## Teaser

When choosing how many eggs to produce, female fruit flies must consider the physical environment and the likely level of competition their offspring will face. Females raised with other females were slower to mate, mated for less time, and laid fewer eggs. However, when choosing where to lay, patchiness of laying sites had a greater impact than social environment: clustered resources attracted more females, who laid on more of the available patches.

## Introduction

### Effects of intrasexual competition

Most individuals experience competition for resources for at least some part of their lifetime. Population density is partly responsible for determining the extent of competition, and the distribution of resources also plays an important role in the spatial and temporal scale at which this density varies (Emlen and Oring 1977; Silver et al. 2000; Noël et al. 2005; Doublet et al. 2019). In particular, more clustered resources (whether food, mates, or nesting/oviposition sites) result in increased encounter rates (Emlen and Oring 1977) and therefore are associated with a greater degree of adult and juvenile competition. Because optimal responses often differ in high- and low-competition environments (Maynard Smith 1979), animals which experience variation in local population density are expected to make plastic adjustments to behavior and physiology in response to prevailing levels of competition to maximize their lifetime reproductive success. To test this prediction, the effect of exposure to conspecific rivals on reproductive investment has been extensively studied, most extensively among males (Gomendio and Roldan 1991; Gage and Barnard 1996; García-González and Gomendio 2004; Klemme and Firman 2013; Droge-Young et al. 2016; Kiss et al. 2019; Kustra et al. 2019; Rowley et al. 2019). *Drosophila* species are an important model organism for understanding when and why such effects are strongest because males are sensitive to the presence of potential competitors and are known to adjust a range of behaviors and reproductive physiological process accordingly, and in ways that vary among species (Bretman et al. 2009; Fedorka et al. 2011; Garbaczewska et al. 2013; Moatt et al. 2014).

Surprisingly less research attention has focused on the equivalent plasticity in females, given the greater contribution than males that variation in their behavior could make to the fitness of offspring (but see Singh and Singh (2014) on density effects on female remating rates, and Battesti et al. (2012) and Sarin and Dukas (2009) on oviposition copying behaviors). Female *Drosophila* are aggressive toward other females, exhibiting a range of behaviors similar to those observed among fighting males (Ueda and Kidokoro 2002; Bath et al. 2017; Chapman and Wolfner 2017). The observation that aggression occurs between female conspecifics, suggests that females may be equally sensitive to the presence of same-sex rivals as males. However, the context in which this aggression manifests is likely to differ compared to males, given the activating neurons are sexually dimorphic (Schretter et al. 2020). Despite female intrasexual aggression being common prior to oviposition, females can also show strong social attraction to conspecifics on food patches (Lihoreau et al. 2016), perhaps because of the facilitation benefits of shared feeding among larvae (Dombrovski et al. 2017). Such attraction of females to laying sites of other females remains to be fully explained, given the trade-off between these benefits and increasing competition, which can lead to cannibalism (Vijendravarma et al. 2013). Tension between competition and cooperative feeding is mediated to some degree by relatedness, with closely related larvae more likely to form cooperative feeding aggregations than unrelated larvae (Khodaei and Long 2020). However, under food restriction, cannibalism is observed even within inbred laboratory strains with high mean relatedness (Vijendravarma et al. 2013).

### Plasticity in oviposition decisions

During oviposition, females can only assess the level of competition their larvae will face based on the number of existing eggs at a patch, or the number of pheromone markings by conspecifics (Malek and Long 2020; Tait et al. 2020). It seems likely therefore that they may also be sensitive to intrasexual encounter rate among adults as a proxy for likely future larval competition. However despite the strong evidence for density and encounter rate effects on male behavior (Bretman et al. 2009; Fedorka et al. 2011; Price et al. 2012; Garbaczewska et al. 2013; Moatt et al. 2014; Hopkins et al. 2019; Churchill et al. 2020), and evidence that females are sensitive to the presence of conspecifics when making oviposition decisions (Malek and Long 2020; Tait et al. 2020), studies of the effect of female encounter rate on subsequent reproductive behavior are rare (Gillmeister 1999). Recently, however, Fowler et al. (2021) demonstrated that both male and female social environment can influence plasticity in various aspects of mating behavior, with interactions between the social environments of the mating pair playing an important role.

In this study, we test whether females respond to the presence of other females during adulthood by subsequently plastically adjusting their egg laying behavior based on the level of competition their larvae might experience. Wild female *D. melanogaster* lay on rotting and fermenting fruits, a naturally patchy, ephemeral, and often unpredictable environment. Females make sophisticated egg laying decisions, including assessing not only the nutritional quality of the resource but also the inter-patch substrate, considering potential energetic costs of larval travel (Schwartz et al. 2012). Given that they lay only one egg at a time (Yang et al. 2008), egg clusters from a single mother are evidence of repeated decisions to lay in the same site. As well as manipulating adult density, we tested to what extent any socially induced plasticity interacts with the physical oviposition environment. We predicted that females would lay fewer eggs per patch on small, isolated patches, and more eggs per patch on clustered patches, because this makes larval travel easier between food sources. Furthermore, we predicted that maternal oviposition decisions would be mediated by the female’s experience of intrasexual encounter rates prior to egg laying, with females from a high-density environment investing more in offspring production in the expectation of high future competition among larvae—either by investing more resources per egg (and so laying fewer eggs overall) or by ovipositing a higher number of less well-provisioned eggs.

## METHODS

All fly rearing and experiments were conducted at 25 °C on a 12-h light:dark cycle (08:00–20:00 h GMT), unless otherwise stated. Stock flies originating from a Canton-S laboratory stock population were housed in 40 mL vials containing 7 mL of a standard agar-based medium (40 g of yeast and 40 g sucrose per liter); hereafter described as standard vials. Approximately 25 *D. melanogaster* were held in each vial, and all vials were pooled and randomly redistributed into new vials every seven days to minimize any within-vial effects of inbreeding, drift, and selective sweeps.

Parental generation vials were set up with a standardized density of six males and six females per vial to ensure food resources were not limiting. Test flies were offspring from these parental vials, collected under ice anesthesia within 6 h of eclosion to ensure virginity, and immediately transferred into treatment conditions.

### Prior experience of competition

Test females were housed in treatment vials for seven days in one of two treatments: singly housed (hereafter “solitary”), or in a group of six females (“grouped”).

### Copulation behaviors

Females were translocated to a new standard vial for copulation with a standardized seven-day old male from a parental vial, which had been housed alone since eclosion. Courtship and copulation behaviors were observed live, and latency to copulate (time from when pair were first introduced until the male successfully mounts the female) and copulation duration (until pair fully separates) were recorded in seconds.

### Oviposition substrate distributions

**Figure 1:**
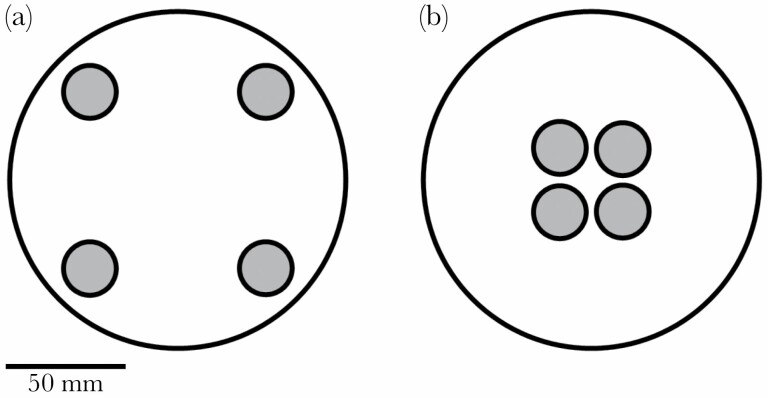
The spatial distribution of oviposition substrates. (a) Dispersed resource distribution treatment: food discs located at four equidistant points around the circumference of the Petri dish. (b) Clustered resource distribution treatment: food discs located in the center of the Petri dish, in a square arrangement with each disc approximately 3 mm apart from adjacent discs.

Females that did not copulate within 90 min of being introduced to the mating vial were excluded from subsequent (egg laying) stages of the experiment. Females which copulated were transferred to individual egg-laying dishes, with oviposition substrate arranged in one of two spatial treatments: dispersed or clustered resources. Petri dishes were 140 mm in diameter and contained four patches of agar-based medium (each 22 mm in diameter, 7 mm depth). For the dispersed treatment, patches were located at four equidistant points around the circumference of the petri dish, at an interpatch distance of 100 mm (Figure 1a). In the clustered treatment, patches were arranged in a square in the center of the Petri dish (Figure 1b). Given that *Drosophila* first-instar larvae travel at an average of 90 µm per second (Heckscher et al. 2012), it would take those on dispersed patches ~18.5 min minimum to travel to a different resource patch, compared to less than 1 min for those on clustered patches. Each patch was placed 3 mm from the edge of the Petri dish, or from other patches, to keep total surface area available for oviposition constant between treatments. The base of each Petri dish was lined with filter paper, to which 10 mL of distilled water was added to prevent food patches from drying out.

This arrangement resulted in four treatments: females from solitary and grouped treatments could lay in either dispersed or clustered resource plates in a fully factorial design. Sample sizes were the following: solitary/clustered resources: 22; solitary/dispersed resources: 22; grouped/clustered resources: 30; grouped/dispersed resources: 29. For the analysis of mating behaviors only, we used an additional six solitary females for which there is no accompanying egg laying data (due to incubator failure).

Females were left to oviposit eggs in these dishes for 18–20 h. Treatment enclosures were placed randomly in three incubators, maintained at 25 °C, under constant light to allow imaging. Each incubator held one Raspberry Pi (www.raspberrypi.org) connected to an 8MP Raspberry Pi Camera module (v2; www.thepihut.com). Frame capture software “raspistill” was used to capture one image every 10 min. For each image, we recorded on which patch of the four food patches the female was found, or if she was not currently on a patch. Once the female had been removed, egg-laying plates were photographed using a digital camera (Panasonic Lumix DMC-FT4) to allow counting of eggs laid per patch.

### Fitness

The plates containing eggs were returned to the incubator, and after 21 days the emerged adults were counted and sexed. During the 21-day emergence period, the filter paper was replenished with 5 mL of water every 3–4 days.

Immediately after females were removed from the plates (18–20 h after introduction), they were given a further seven days to lay any remaining eggs in a standard vial, before being removed for wing size measurements to be taken. Number of male and female offspring were counted 14 days later.

### Statistical analysis

All statistical analyses were conducted in R v4.0.3 (R Core Team 2019). We tested the effects of treatment on response variables using mixed effects models with the appropriate error distribution (binomial error for egg presence/absence, negative binomial error distribution for the overdispersed egg and offspring number, Gaussian for mating latency and duration) with the functions in packages lmerTest (Kuznetsova et al. 2017), and lme4 (Bates et al. 2015). This approach allowed us to fit vial identity as a random effect to account for shared housing of females in grouped treatment. However, in all models, the variance component for vial was estimated at non-significantly different from zero leading to a singular model fit, so we re-ran these using (generalized) linear models with the appropriate error distribution: binomial for the egg presence/absence data, quasi-Poisson error for the egg and offspring number models to account for overdispersion, and Gaussian for mating duration. We used a Kaplan Meier survival analysis to analyze mating latency data to account for individuals that did not copulate.

We analyzed whether the number of patches that a female chose to lay on was influenced by either prior housing or oviposition substrate treatment using Chi-squared tests. Although this does not allow us to treat the effect of shared housing as a random effect, this was not a significantly confounding factor in any of the previous models.

## RESULTS

### The impact of prior exposure to density on female mating behaviors

**Figure 2:**
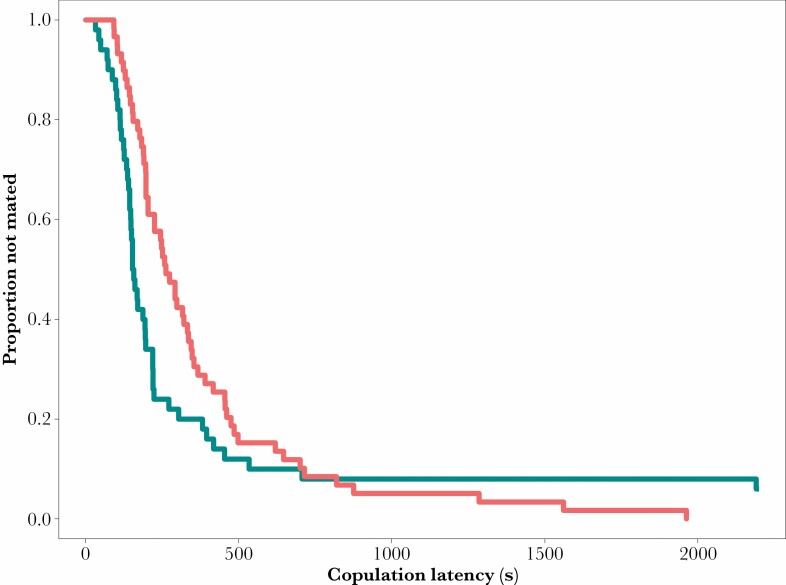
Survivorship curves of latencies of females to copulate. Blue curve—solitary-housed females; red curve—group-housed females.

All females were courted, and all group-housed females, and all but 3 out of 60 single-housed females copulated. However, group-housed females were at least 2 min slower to copulate than solitary females (latency to mate, solitary: 235 ± 47 s SE; group-housed: 373 ± 45 s SE; Kaplan Meier survivorship: χ ^2^ = 9.67, df = 1, *P* = 0.00187; Figure 2).

**Figure 3:**
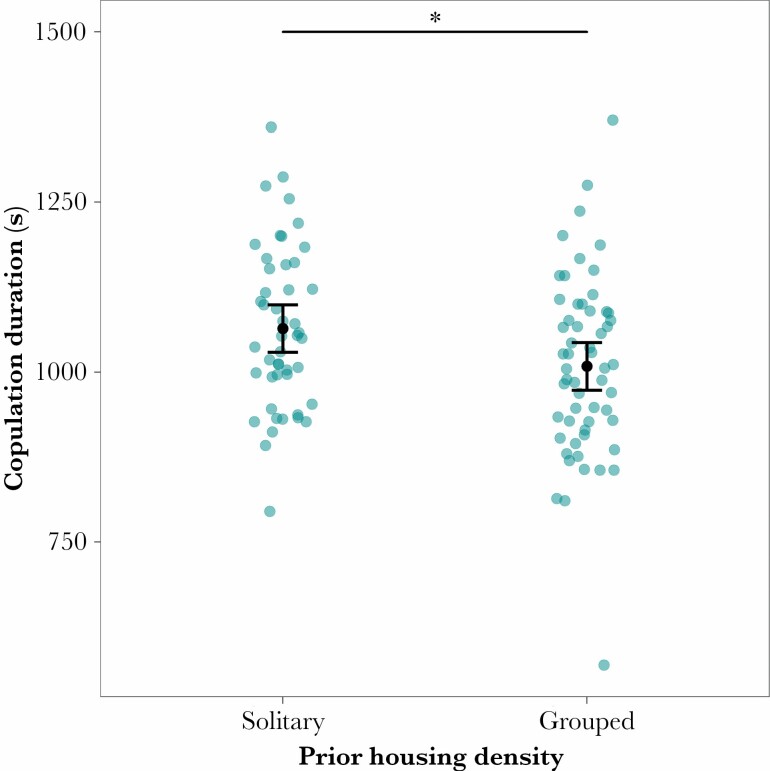
Solitarily housed females mated for longer than those previously housed in groups. Means (black dot) and 95% confidence intervals of copulation duration are shown in seconds. The difference in means remains significant after removal of the low outlier in the group-housed treatment. Note that the analysis was performed on logged data, but untransformed values are presented here.

As well as being slower to start copulating, co-housed females’ mating duration was an average 55 s less than that of solitary females (linear model: log_10_(mating duration): *F*_1,102_ = 4.97, *P* = 0.0255; Figure 3). This difference remained significant after removal of an outlier in the group-housed group (linear model; *F*_1,101_ = 4.10, *P* = 0.0418; Figure 3).

### Effects of prior housing density and resource spatial distribution on oviposition and fitness

Female oviposition behavior could be influenced by two treatment conditions: prior housing (either group-housed or solitary) and/or current oviposition landscape (either clustered or dispersed food patches). Of these, clustered resources led to a significantly higher proportion of females laying in the 18–20 h given, than dispersed resources (general linear model with binomial errors: χ ^2^ = 4.88, *P* = 0.0272; solitary odds ratio = 1.429; group-housed odds ratio = 1.269; Table 1). However, there was no significant interaction between these variables in terms of whether eggs were laid or not (χ ^2^ = 1.24, *P* = 0.265), and although the percentage of females laying eggs was reduced among group-housed females, prior housing treatment had no significant effect (χ ^2^ = 2.74, *P* = 0.098; Table 1).

**Table 1:** The effect of competition and resource distribution on the percentage of females which laid at least one egg in the given 18–20 h

		Prior housing treatment	
		Solitary	Group housed
Oviposition resource distribution	Clustered	90.9%	70.0%
	Dispersed	63.6%	55.2%

**Figure 4:**
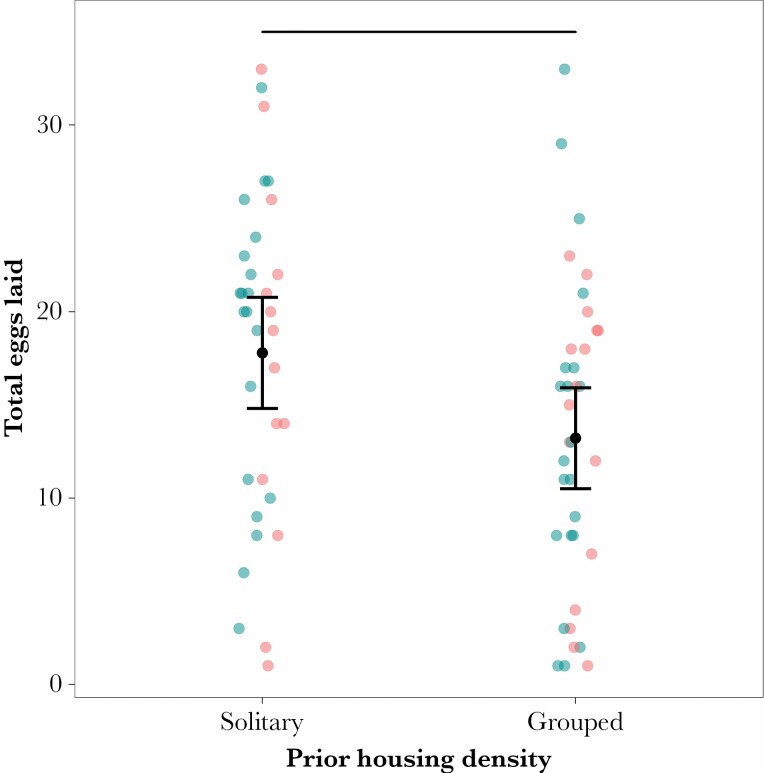
Prior housing density significantly influenced how many eggs were laid in the 18–20 h oviposition window, with females from a solitary background laying more eggs than those that had previously been group housed. Resource distribution, by comparison, had no significant effect on laying rate (blue: clustered resources; red: dispersed resources). Means (black dot) and 95% confidence intervals are shown for the two housing densities. Further detail can be found in the supplementary information.

Among those females that laid eggs, the effect of these two treatments was reversed. While prior housing had no effect on whether eggs were laid, it did significantly influence clutch size, with group-housed females laying 22% fewer eggs than those from a solitary background (solitary: 18 ± 2 eggs; group housed: 14 ± 1 eggs; generalized linear model with quasi-Poisson errors *F*_1,69_ = 5.106, *P* = 0.027; Figure 4). And while food patchiness significantly affected the probability of laying eggs, it had no equivalent effect on egg number (*F*_1,68_ = 0.073, *P* = 0.788). Again, there was no interaction between treatments (*F*_1,67_ = 0.076, *P* = 0.783) in this model.

Surprisingly, these effects of prior housing and egg-laying resource distribution did not lead to any difference between treatments in the proportion of eggs that survived to adulthood (competition: *F*_1,34_ = 0.115, *P* = 0.737; oviposition substrate: *F*_1,63_ = 0.0450, *P* = 0.833; treatment interaction: *F*_1,63_ = 3.59, *P* = 0.0629). Neither treatment (competition: *F*_1,85_ = 2.145, *P* = 0.147; oviposition substrate: *F*_1,85_ = 0.0176, *P* = 0.895), nor the interaction (*F*_1,85_ = 0.235, *P* = 0.629), affected the sex ratio of offspring produced.

### Oviposition distribution and fitness among varying resource distributions

**Figure 5:**
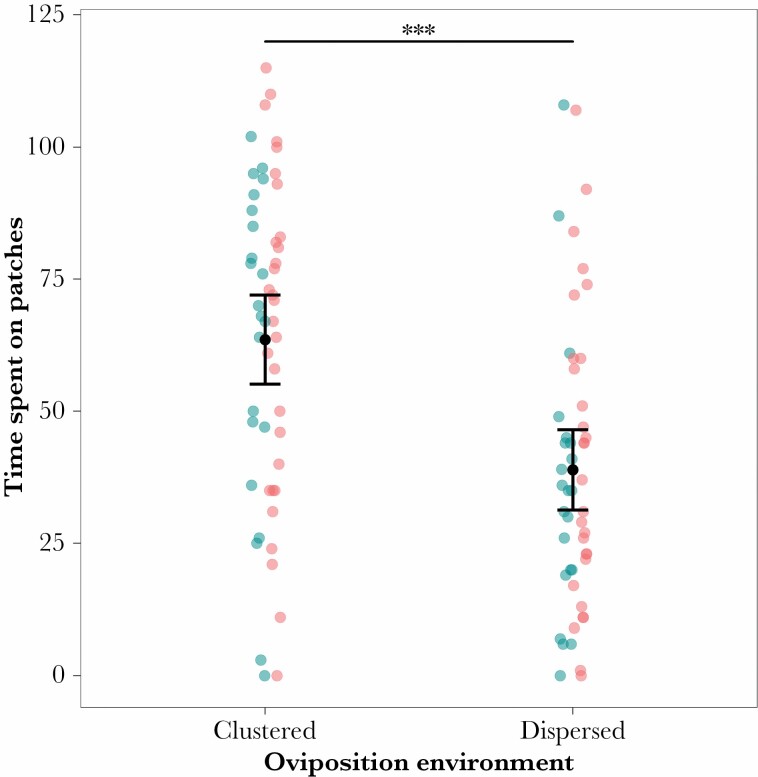
Females house on clustered oviposition substrates spent more time visiting those resources (measured as the number of images in which the female was observed on a food patch). Prior housing density had no significant effect on laying rate (blue: solitary females; red: grouped females). Means (black dot) and 95% confidence intervals are shown for the two oviposition substrate distributions. Further detail can be found in the supplementary information.

Females on dispersed resources spent less time on the food patches (34.2% of time on resources) than those on clustered resources (56.1%: raw number of records on food patches, linear model: *F*_1,99_ = 18.64, *P* << 0.001; Figure 5). Prior housing treatment had no equivalent effect (*F*_1,99_ = 0.321, *P* = 0.572), and there was no interaction between the two treatments (*F*_1,99_ = 0.155, *P* = 0.695).

**Figure 6:**
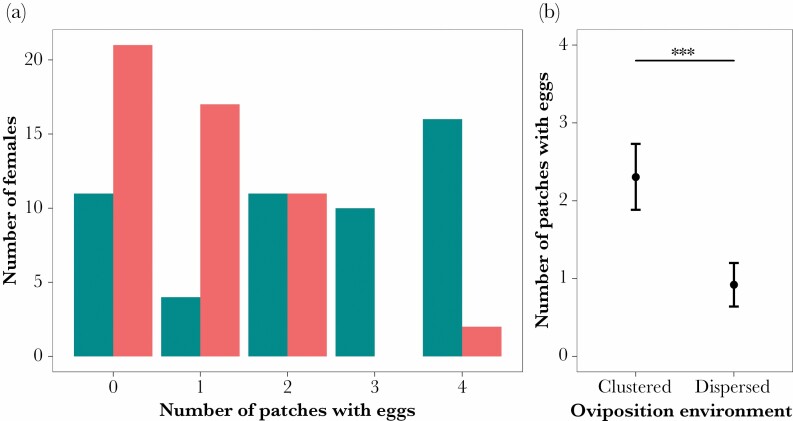
Females laid eggs on more of the available patches when these patches were clustered than when they were dispersed. (a) Bars show the number of females which laid on either zero, one, two, three or all four available oviposition patches in two different spatial arrangements. Blue bars—clustered resources (*N* = 52 laying females); red bars—dispersed resources (*N* = 51). (b) The mean number of patches laid on by females in clustered and dispersed oviposition substrate distributions. Means (black dot) and 95% confidence intervals are shown for the two oviposition substrate distributions.

There was no effect of housing treatment on the number of patches on which eggs were observed (χ ^2^ test: χ ^2^ = 5.44, *P* = 0.245), but females laid eggs on more of the available patches when these patches were clustered (x̄ = 2.31; mdn = 2.5) than when they were dispersed (x̄ = 0.92; mdn = 1.0; χ ^2^ = 32.06, *P* < 0.001; Figure 6).

### Oviposition distribution on individual resource patches

Across all treatments, females were more likely to lay on the edge of patches than on the top surface (sum of eggs on sides vs. top of four food patches; paired *t*-test, *t* = −6.426, *P* < 0.001); and this pattern was consistent within each of the four treatment combinations (all *P* < 0.013).

### Post-treatment egg production

Of the 101 females given the opportunity to lay in both Petri dishes and vials, 70 laid in both, 10 laid in neither, 20 laid none in the Petri dishes but did lay in the vials, and only 1 laid in the Petri dishes but none in the vials. The probability of laying in vials was thus significantly positively influenced by whether or not eggs had been laid in the Petri dish (GLM with binomial errors: χ ^2^ = 20.8, *P* < 0.001), but not by either treatment (both *P* > 0.5). The number of offspring produced across both Petri dish and vial was not significantly affected by either prior housing density (χ ^2^ = 14.50, *P* = 0.305) nor resource distribution in the Petri dish (χ ^2^ = 14.22, *P* = 0.309).

**Figure 7:**
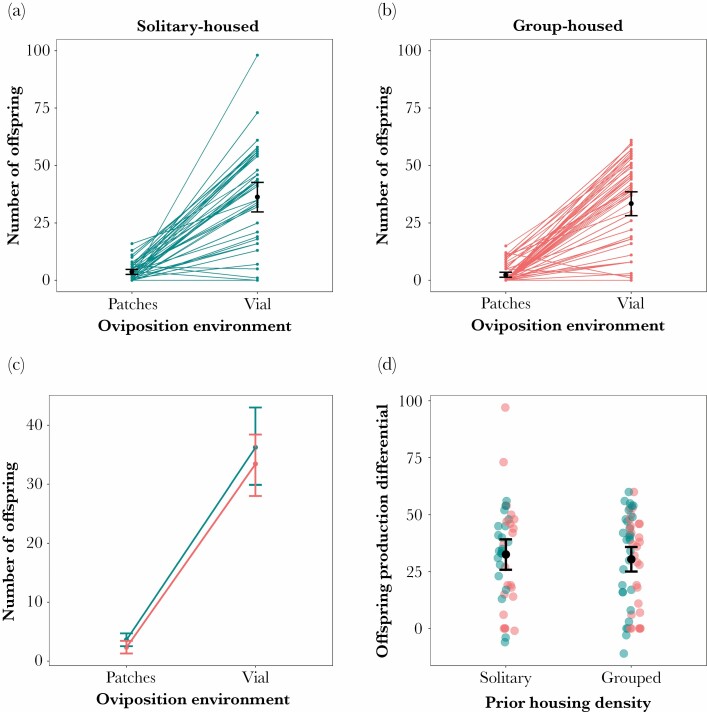
Number of adult offspring produced by females in patchy oviposition environments and standard vials. Females were given one day to lay on resource patches, before being given a further 7 days to oviposit remaining eggs in a standard vial. (a) Number of offspring produced by females previously housed in solitude. Means (black dot) and 95% confidence intervals are shown for the two oviposition substrate distributions. Lines connect offspring number from the same female. (b) Number of offspring produced by females previously housed in groups. Means (black dot) and 95% confidence intervals are shown for the two oviposition substrate distributions. Lines connect offspring number from the same female. (c) The mean and 95% confidence intervals for the two patchiness treatments (blue: solitary females; red: group-housed females). (d) The per-female difference in the number of offspring produced in the patchy environments compared to standard experiment vials. Positive values indicate that more offspring were produced in the standard vials.

To better understand the impact of treatment on the production of offspring, in each group-housing treatment we calculated the difference in the number of adult offspring produced on the patches and in standard vials—see Figure 7. We found no effect of prior housing density (*F*_1,24_ = 0.105, *P* = 0.749) or oviposition resource distribution (*F*_1,78_ = 0.203, *P* = 0.654).

## Discussion

### The impact of prior exposure to density on mating behaviors

Females that had been housed in groups prior to mating took longer to start copulating and copulated for a shorter duration than females maintained alone since emergence. Assuming that both groups of females have some control on mating durations (Spieth 1974; Mazzi et al. 2009), these behaviors suggest a greater reluctance to mate among females from a group housed background. This matches findings from previous studies, which demonstrate that females are often more choosy in higher-density mixed-sex populations (Lehmann 2007; Willis et al. 2011; Atwell and Wagner 2014; Scott et al. 2020), where the risks of remaining unmated are lower and there is less pressure to mate with the first available male.

Here, we interpret the delayed start of copulation and the shorter mating duration as indicators of choosiness—females from a grouped background showed lower willingness to mate with the first available male, and mated with him for less time, in expectation of future mating opportunities. It is true that females in our grouped treatment encountered only other females, but they may use female encounter rate as evidence of generally higher population density, and therefore a greater likelihood of encountering multiple males. However, the opposite expectation is also plausible—higher female encounter rate without encountering males may lead to an expectation of low male presence. The observed decrease in willingness to copulate at higher female densities could instead be due to a trade-off with previously increased energy expenditure in social behaviors experienced by group-housed females, or group-housed females could be slower to identify male conspecifics than those that have not previously been exposed to consexuals.

In contrast to these explanations, females housed in solitude were less likely to successfully copulate than those housed in groups. It is possible that males viewed grouped females as more attractive (as they could sense the pheromones of multiple females (Marcillac et al. 2005; Dweck et al. 2015), but no evidence of this was found in likelihood to court, as all females were courted. However, given no measurements of courtship effort were recorded it could be that males courted solitary females with less vigor, and so that females were less likely to accept copulation attempts. In wild great tits, Firth et al. (2018) showed that bold individuals paired with their mates more quickly, but then proceeded to have a decreased chance of successful matings. It is possible that we have observed a similar effect here; with those that are less-choosy (i.e., quicker to court) being more likely to fail to achieve copulation.

It is interesting that these density effects on females are the opposite to those demonstrated for males exposed to consexual rivals prior to mating, which stimulates more extended copulation durations in *D. melanogaster* and a number of other species (García-González and Gomendio 2004; Bretman et al. 2009; Flay et al. 2009; Klemme and Firman 2013). Male responses are interpreted as a reaction to a perceived increased risk of sperm competition, which males can best counteract by mating for longer—perhaps to increase the quantity of sperm transferred (Simmons and Parker 1992; Engqvist and Sauer 2003), but also possibly as a form of mate guarding (Vitta and Lorenzo 2009). Because our measure of mating latency includes the time taken for males to initiate courtship, variation in mating latency might also be influenced by males’ reduced willingness to court females from group housed backgrounds. Although there is evidence for females influencing copulation duration, it is also clear that males can affect this trait. So, an alternative explanation for the increased mating latency and reduced copulation duration observed is that this variability is due to male rather than female behavior. Males paired with previously group-housed females may have detected apparent high density of other females via pheromones remaining on the focal female, given that males are known to be sensitive to the pheromones of other males carried on females (Friberg 2006). This means that, although the responses of males to female density have not yet been explored in this species, the lengthened latency to copulate and subsequent shorted copulation duration could be due to males anticipating additional mating opportunities, influencing how much they invest in the focal female.

### Effects of prior housing density and resource spatial distribution on oviposition decisions and subsequent fitness effects

A higher proportion of females housed on clustered resources laid eggs, compared to those housed on dispersed oviposition resources. Those on clustered resources also laid on more of the available patches than those on dispersed resources. Clustered resources within a given area reduce the search time for females, meaning that they have more time available to lay eggs. This is supported by our data on the time spent on patches: females on the dispersed medium spent less time on patches than females housed on clustered resources. However, the observation that some females did not lay at all, particularly on the dispersed patches, suggests that dispersed patches may also be perceived as a less valuable resource than clustered. This may arise from the observation that females consider the degree to which larvae will need to travel when choosing oviposition sites (Schwartz et al. 2012), where clustered patches better facilitate social aggregation in larvae, perhaps allowing for more efficient cooperative feeding (Dombrovski et al. 2017; Khodaei and Long 2020).

There may be physical environmental explanations for the difference in laying success on clustered versus dispersed resources. Like others, we found that females laid more eggs on the edge of the resource patches than on the top surface (Moore 1952; Chess and Ringo 1985). Although we did not control for the fact that patch side area is greater than patch top area, the total area available for laying is the same under both resource distributions. The number of sheltered edges is increased in clustered resources, and as *Drosophila* preferentially lay on the edges of resources, this increase in sheltered edges could be driving the preference for clustered egg-laying patches. Additionally, small patches of food are likely to dry out much more rapidly than larger patches, and clustering may help to mitigate this effect as well. A desiccated food resource will inevitably limit larval survival, providing another explanation for females preferring this arrangement of patches. Further work is necessary to discriminate between these explanations for female behavior.

Although the distribution of egg-laying patches influenced the probability of eggs being laid and the number of patches laid on, this physical environment had no effect on the number of eggs laid. By contrast, we found that prior housing treatment was important: group housed females laid fewer eggs irrespective of egg-laying environment, a result that also appears to be present in the data of Fowler et al. (2021). Females engage in energetically expensive aggressive interactions with their consexual rivals in this species (Ueda and Kidokoro 2002; Bath et al. 2017). These aggressive interactions could lead to a trade-off in which group-housed females have less energy available for oviposition. In addition, when females oviposit in the presence of rivals, they copy their oviposition behaviors to reduce costs associated with sampling the available substrate (Malek and Long 2020). In the absence of such information, females may have been slower to choose where to deposit their clutch. Finally, females from the group housed treatment are likely to anticipate a high level of competition for their offspring. This may have caused them to reduce the size of the clutch, perhaps also increasing the size of eggs, or the quality of provision, to improve their competitive advantage. However, although we did not measure egg size, we found no evidence to suggest there was a trade-off in the quality of eggs laid, or female investment per egg, as we found no treatment effects on the number of successfully eclosed adults from these eggs. This observed decrease in clutch size, suggests the alternative prediction of an increased clutch size under high density was not true here. It is also possible that any such fitness effects of egg investment were absent because of the benign laboratory conditions (e.g., ad libitum food and constant temperature). Future work should test for fitness effects in a more stressful environment and over subsequent generations.

Fitness effects may also have not been detected because our experimental design required the removal of the females before they had completed oviposition of all fertilized eggs: this was necessary so that the location of oviposited eggs could be recorded, as larvae hatch after 22–24 h at ~25 °C (Fernández-Moreno et al. 2007; Markow et al. 2009). Had females been given the opportunity to continue ovipositing on the patchy resources, their choice of egg location could (eventually) have impacted overall fitness. Equally importantly, a benefit of clustering oviposition sites is likely to arise in the presence of nonrelated conspecific larvae, meaning that fitness benefits may have been observed had the other females been given the opportunity to oviposit on the same resources.

In this study, we have demonstrated that female density has significant effects on mating and egg-laying behavior. Females from group-housed conditions are slower to accept mating and mate for less time, which we suggest is related to future opportunities to mate, and lay fewer eggs, probably due to competition for their larvae that the mothers infer from their own conspecific density as adults. Surprisingly however, although the physical arrangement of egg-laying patches has significant effects on oviposition behavior, with clustered resources being preferred over dispersed ones, there is no interaction between this physical stimulus and the social stimulus of perceived population density. Although reproduction-related effects of density are already well known in males (Bretman et al. 2009; Fedorka et al. 2011; Garbaczewska et al. 2013; Moatt et al. 2014), equivalent study in females has so far been lacking. We have demonstrated that social environment has profound effects on females too. The way that the social environment affects the behavior of females is of particular interest, especially given the more pivotal role of female behavior in affecting the demography, persistence, and evolvability of populations, especially where multiple mating occurs.

## Supplementary Material

arab105_suppl_Supplementary-Material

## Data Availability

Analyses reported in this article can be reproduced using the data provided by Churchill et al. (2021).
