# Supplementary material for: Social and physical environment independently affect oviposition decisions in Drosophila
Source: Behav Ecol. 2021 Sep 22;32(6):1391–9. doi: 10.1093/beheco/arab105 (PMC8691557; doi:10.1093/beheco/arab105)
Supplement: arab105_suppl_Supplementary-Material [file arab105_suppl_supplementary-material.docx]

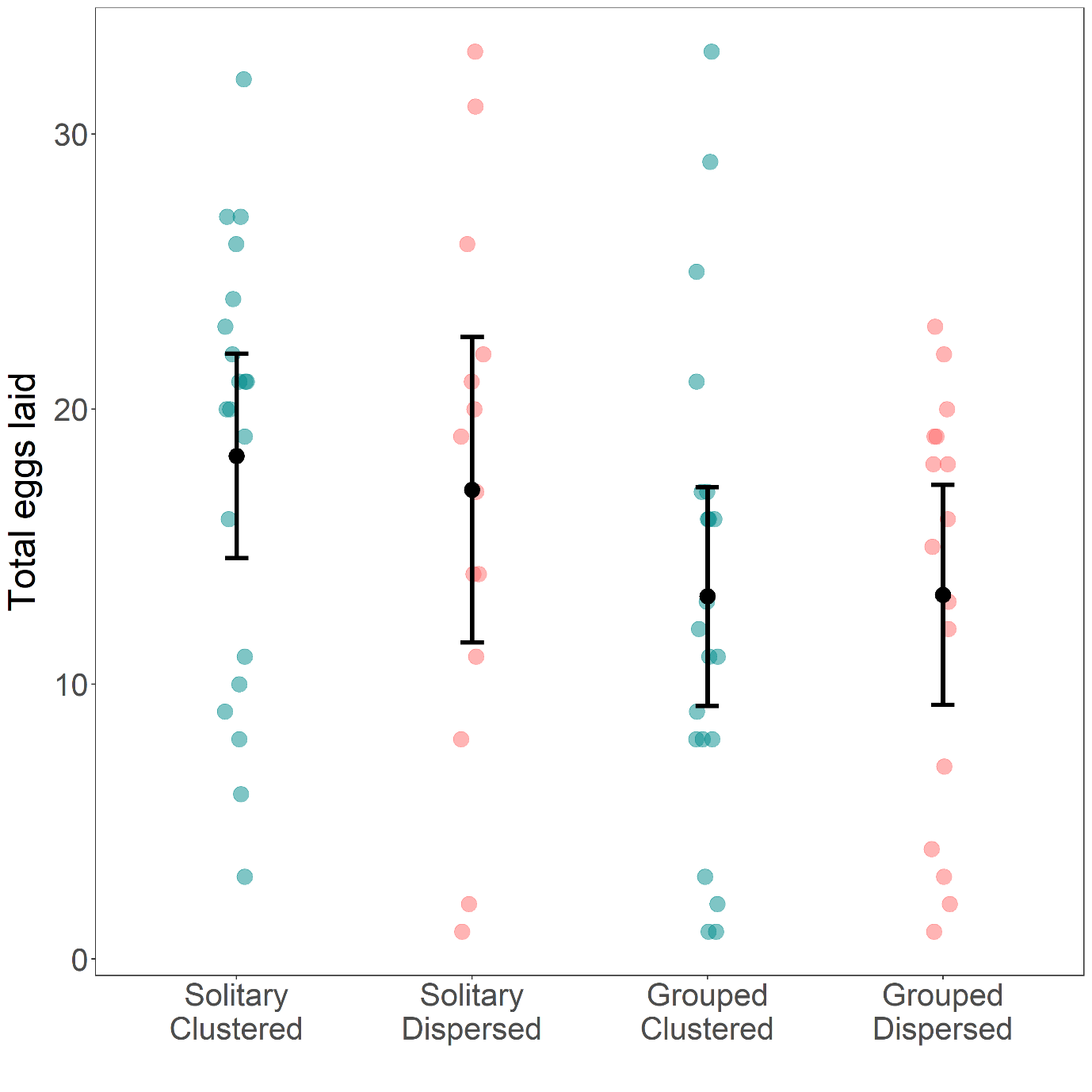


Figure 8. The effect of prior housing density and oviposition substrate distribution on the total number of eggs laid by the female. Means (black dot) and 95% confidence intervals are shown for the four treatments.


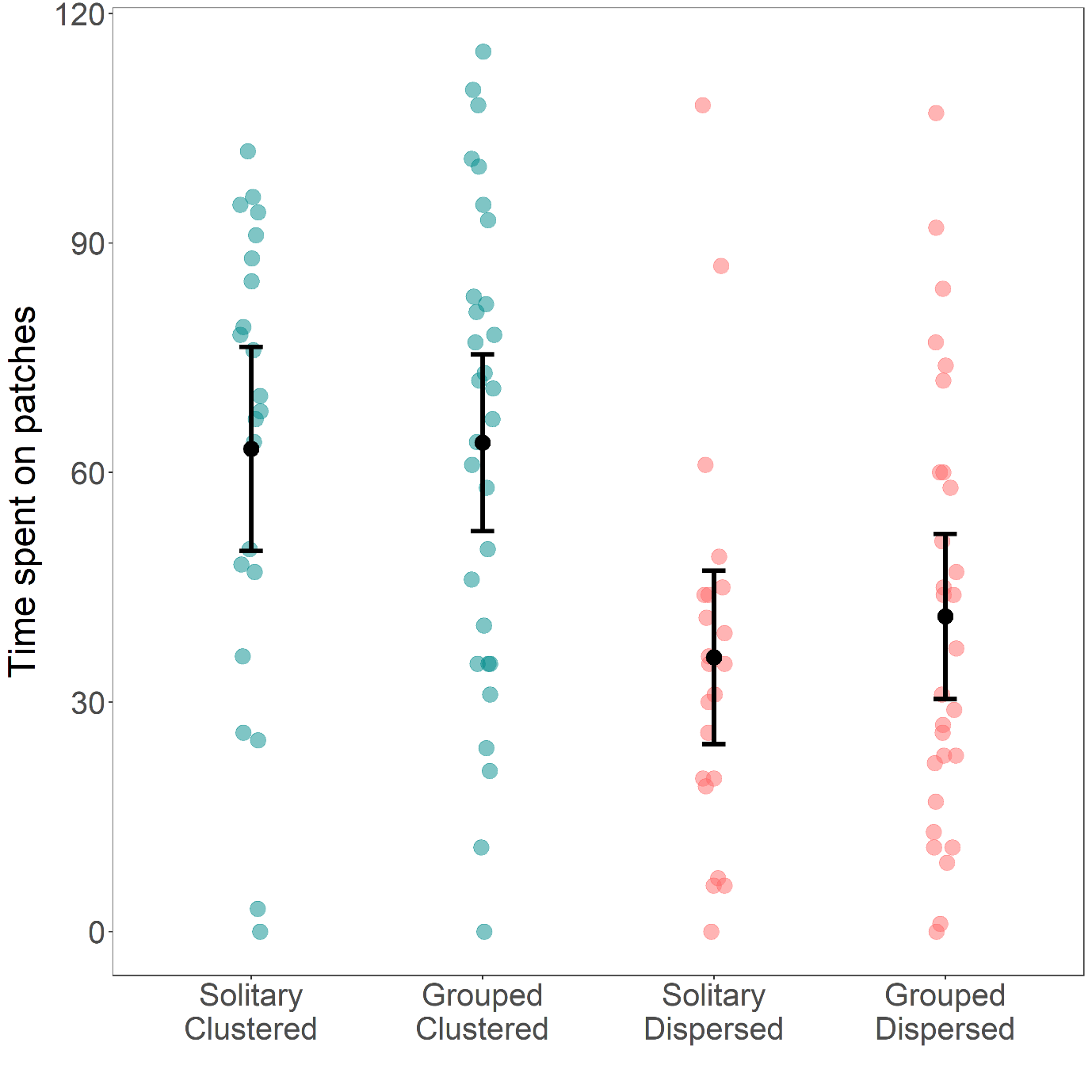


Figure 9. The effect of prior housing density and oviposition substrate distribution on the time females spent on the available patches (measured as the number of images in which the female was observed on a food patch). Means (black dot) and 95% confidence intervals are shown for the four treatments. Order of treatment types differs the Fig. 8 for ease of comparison of the significant differences shown in Fig. 5.
